# Substance use Specificities in Women with Psychosis: A Critical Review

**DOI:** 10.2174/1570159X21666221129113942

**Published:** 2023-07-10

**Authors:** Francesc Casanovas, Francina Fonseca, Anna Mané

**Affiliations:** 1 Institut de Neuropsiquiatria i Adiccions (INAD), Parc de Salut Mar, Barcelona, Spain;; 2 Hospital del Mar Medical Research Institute (IMIM), Barcelona, Spain;; 3 Department of Medicine and Life Sciences (MELIS), Universitat Pompeu Fabra, Barcelona, Spain;; 4 Centro de Investigación Biomédica en Red, Área de Salud Mental (CIBERSAM), Madrid, Spain

**Keywords:** Women, psychosis, schizophrenia, dual disorders, substance-use disorders, substance-induced psychosis

## Abstract

**Background:**

Women with schizophrenia or other psychotic disorders differ from male patients in many respects, including psychopathology, prognosis, disease course, and substance use comorbidities. Most studies performed to date to investigate the association between drug use and psychosis have not evaluated gender differences, although this has started to change in recent years.

**Methods:**

We briefly summarize the available evidence on gender differences in drug use and substance use disorders (SUD) in psychotic patients during the early phases of the psychotic illness and during the course of schizophrenia.

**Results:**

Substance use and SUD are both less prevalent in women, both in the general population and at all phases of the psychotic spectrum. Some studies suggest that SUD may be under diagnosed in female patients, in part due to their more vulnerable profile. Substance use, especially cannabis, may more negatively impact females, especially on the disease course and prognosis. The available data suggest that it may be more difficult to treat SUD in female patients with schizophrenia, which could negatively impact prognosis.

**Conclusion:**

Women with concomitant psychotic illness and SUD comprise a highly vulnerable subgroup. This should be considered when selecting the treatment approach, especially in the early phases of the illness, to ensure better outcomes.

## 1 INTRODUCTION

The use of psychoactive substances is associated with the etiology, course, and prognosis of psychotic disorders, particularly schizophrenia-related disorders [1]. Patients with schizophrenia are a highly heterogeneous group. In recent years, several studies have suggested that gender differences could partially explain some of this heterogeneity, although more research is needed [2]. In mental disorders, a gender perspective must consider not only biological differences between males and females but also the social and psychological consequences of gender. The National Institutes of Health Office of Research on Women’s Health defines sex as the biological differences between females and males (chromosomes, sex organs, hormones) [3]. By contrast, the term gender refers to socially-determined roles and behaviors, which can-and do-vary across cultures and time.

Despite the importance of gender perspectives in psychosis, most research conducted to date on psychosis and comorbid substance use has not specifically investigated the role of gender. Gender has been mostly studied as a covariate in the analyses, but not as the central hypothesis of the study [4, 5]. However, more recent studies have specifically focused on gender differences [6, 7].

Although it has been classically reported that women have a lower prevalence of substance use, the clinical picture may be more severe and the prognosis worse than in men [6]. In this context, the aim of the present critical review is to summarize the main findings in the literature on the specificities of substance use in women with psychosis. We include the latest findings on different phases of the psychotic disorder, ranging from prodromic phases to chronic schizophrenia, with a special focus on substance-induced psychosis and other types of psychosis.

## 2 SUBSTANCE USE AND PSYCHIATRIC COMORBIDITY: BASIC CONCEPTS

In cohort studies, females have historically accounted for a smaller percentage of drug users, suggesting that substance use disorder (SUD) is less prevalent among women [8]. However, several different factors may influence the lower reported prevalence of SUD among women. First, most of the studies that have assessed gender differences in SUD are not community-based, which means that help-seeking behavior, which is lower in women, could affect the findings [9]. Several other factors may also prevent women from accessing clinical care, including social stigma, feelings of shame, denial, and the negative impact of SUD on maternity [8]. In addition, research findings suggest that there may be a bias against diagnosing female patients with SUD given that women receive half as many SUD diagnoses as men, even though the proportion of positive screening tests for drug use is similar [10]. All of the aforementioned factors could lead to the under diagnosis of SUD in female patients. Nonetheless, this gender gap in the prevalence of SUD has decreased in recent years [8], which may be explained by an increase in substance use among women and/or, an increase in help-seeking behavior among women.

The most common substances used by men are cannabis, stimulants (cocaine, amphetamines), and hallucinogens. By contrast, prescription drugs, such as sedatives and opioids, are more common in women. In the general population, the drugs most commonly used by women are alcohol, tobacco, and cannabis [11].

Another key element in terms of the specificities of SUD in women is the course of the addiction. Although women tend to start using at a slightly older age than men, they present an accelerated progression from their first use of a substance to the onset of dependence because they increase their frequency of use faster than men. This is a phenomenon known as the “telescoping effect” [12], and it has been described in women for opioids, cannabis, alcohol and cocaine [6]. In fact, women are more likely to present at healthcare facilities with more severe somatic and psychiatric comorbidities [13, 14]. Moreover, a higher proportion of women with SUD are diagnosed with the dual disorder (DD) [8, 15]. It is important to note that most studies performed to investigate DD have involved patients treated at an Addiction Treatment Unit using instruments developed for other common disorders, such as anxiety and depressive disorders, rather than for psychotic disorders. Furthermore, most of the studies on DD performed to date have mainly involved male patients [16].

Tirado *et al*. (2018) evaluated psychiatric comorbidity in female intravenous drug users from five different European regions [17]. The diagnostic instrument used in that study was the dual diagnosis screening interview (DDSI), which is commonly used to assess comorbid SUD and mental disorders. Even though numerous studies have reported a high prevalence of SUD in patients diagnosed with a psychotic disorder [18], Tirado and colleagues did not apply the psychosis module of the DDSI in that study, underscoring the scant research on the presence of psychotic symptoms in women with SUD.

Recently, Ferrer *et al.* (2020) used the DDSI, including the psychosis module, in a group of patients with SUD admitted to a general hospital (for any reason) who were attended by a consultation-liaison addiction unit [19]. Interestingly, the dual diagnosis was higher in women than men (53.8% *vs.* 32.7%), although this difference was not statistically significant (*p* = 0.156). Most of the psychiatric comorbidities were more common in women, although without reaching statistical significance, probably due to the small sample size (13 female and 55 male patients). Psychosis was also more common in women (30.8% *vs.* 19.1%; p = 0.296), and although not statistically significant (again due to the small sample size), this finding underscores the importance of exploring psychotic symptoms in women with SUD, even if psychosis is not the main reason for consultation. SUD has been associated with a higher risk for psychosis, with one study reporting that women had a significantly greater risk than men (odds ratio: 7.04 *vs*. 4.24, respectively) [20], further underscoring the importance of assessing for psychotic disorders in women with SUD. Finally, Ferrer *et al.* (2020) found that women had greater severity of dependence and lower perceived quality of life [19], findings that indicate that women with SUD tend to be more severely ill.

## 3 WOMEN WITH SCHIZOPHRENIA AND SUBSTANCE-USE DISORDERS

In patients with schizophrenia, substance use has a negative effect on the course of the psychiatric illness and is associated with a higher risk of relapse, more hospitalizations, and greater use of health care services [1]. Substance use is also associated with increased mortality, with one study finding that it reduced life expectancy by 13 to 15 years [21]. In a recent study based on nationwide cohort databases from two Scandinavian countries, the prevalence of SUD was associated with a higher risk of psychiatric hospitalization and mortality rates, especially secondary to suicide attempts or other external causes [18]. In that study, the most common SUDs involved multiple drugs, followed by alcohol and cannabis. The use of more than one drug is common in patients with schizophrenia, which makes it difficult to evaluate the specific influence of a single substance [22]. Lahtenvuo and colleagues also found that the majority of patients in all three study groups were males [18], in line with other reports [23]. Furthermore, in that study, the prevalence of SUD among patients with schizophrenia was high (≈ 30%), although lower than in older studies [24, 25]. As those authors observed, SUD may be under diagnosed in patients with schizophrenia, perhaps because psychiatrists tend to avoid making an official diagnosis of addiction in patients with schizophrenia to ensure adherence to follow-up. Some studies suggest that it is more common to underreport drug use in patients with schizophrenia than in other severe mental disorders [26], especially during the acute phase of psychosis [27]. Patients with schizophrenia may fear the double stigma (psychotic illness and addiction); this is especially true for women, due to the repercussions in terms of social stigma, self-stigma, and social and family consequences. Although female patients with comorbid schizophrenia and SUD have more social contacts and fewer legal problems than men, they also tend to have more problems related to victimization and medical illness [28]. Bahorik and collaborators (2014) evaluated the discrepancies between self-reported drug use and urine tests in a subsample of 1042 patients with schizophrenia [29] included in the CATIE [30]. In that sample, self-underreporting of drug use was significantly higher in women (66.7% *vs.* 54.8%, respectively; *p* = 0.027), and also more common in African-American *vs*. Caucasian patients (63% *vs.* 54%, *p* = 0.047). Interestingly, on the logistic regression model, the most consistent predictor of underreporting was greater neurocognitive impairment; by contrast, racial status was only associated with underreporting of cannabis use. Gender had no significant effect on underreporting.

Kozack *et al.* (2021) recently reviewed cannabis-use disorder and psychiatric comorbidity, finding that men were more likely to present a dual diagnosis of the cannabis-use disorder and any other mental illness [7]. The risk was especially high for patients with schizophrenia, who had a three-fold higher risk for a cannabis-related SUD.

In most regions of the world, female patients with schizophrenia have a lower baseline rate of substance use than males [31]. Despite the higher prevalence of SUD in male patients, data from some studies suggest that gender differences in terms of the prevalence of substance use may decrease over time [32]. Although women tend to have better outcomes in terms of functionality, employment, and social relations, these outcomes tend to converge over time, perhaps due to increased substance use among women. In this regard, cannabis use in women could have a greater negative impact on long-term outcomes in schizophrenia [33-35] because women are less likely than men to reduce their use of cannabis over time [6]. Given these data, it seems clear that it is important to reduce substance use in all patients (both males and females) with schizophrenia rather than mainly targeting male patients, as some authors have proposed [36].

Although no association between alcohol use and age of psychosis onset has been described [22], alcohol use in these patients has been associated with worse outcomes, including an elevated suicide risk [37]. In the general population, alcohol misuse tends to develop earlier in men and later in women, although symptom severity is similar. By contrast, among male and female patients with schizophrenia who use alcohol, the clinical characteristics are similar in terms of age at onset, the severity of alcohol abuse, and months of abuse [28]. Nevertheless, the prevalence of alcohol use is lower among women with schizophrenia than among men [31, 38].

Compared to the general population, the proportion of patients with schizophrenia who smoke cigarettes is high, ranging from 60% to 90% [1]. There is a strong association between smoking and schizophrenia. Studies have shown that smoking is a robust predictor of future risk for schizophrenia among both genders [39]. In addition, men with psychotic-spectrum disorders are more likely to present comorbid tobacco use disorder [40]. Among females, tobacco use in patients with severe mental disorders is almost three times higher than in the general population [41, 42], especially among older women. This association is especially concerning given the somatic comorbidities in women with schizophrenia, such as gynecological cancers, with notably elevated incidence and mortality rates [43, 44], which are also influenced by tobacco use.

Although the increased prevalence of smoking has been classically thought of as a consequence of schizophrenia, current epidemiological studies suggest that it should be considered as a risk factor for this disorder [45]. Cigarette smoking is associated with different pro and anti-inflammatory mediators [45]. Smoking represses part of the antioxidative response leading to cellular damage that has been related to the etiopathogenesis of normal aging, neurodegenerative disorders and major psychiatric disorders [46, 47]. The brain detects and defeats oxidative stress through a complicated network that involves the expression of vitagenes, such as HO-1 [48], genes involved in preserving cellular homeostasis during stressful conditions [49]. The diathesis-stress model of schizophrenia defends that dysregulation of adaptative stress responses lowers resilience and increases vulnerability to a psychotic disorder. Resilience is the process of adapting and coping with adverse life events including chronic stress, physical or sexual abuse, negligence, or parental mental illness [50]. Individual and familiar resilience factors have also been associated with the onset and course of a SUD [51]. The type of stressful event and the social context of the victim can modify the resilient response [52]. Female patients present a higher social premorbid functioning at early phases of psychosis [53], which could benefit their resilient response and could partly explain the better outcomes at the onset of the disease.

There is little research aimed at knowing the efficacy of interventions for dual psychotic disorders in women. Considering that atypical antipsychotics have shown the best results in treating patients with psychosis and a comorbid SUD [54, 55] its use in women with dual psychotic disorders could be specially indicated considering the predominance of affective symptoms, for which this type of drug presents a better profile. In this sense, the concomitant use of mood stabilizers should also be considered.

In short, in patients with schizophrenia, substance use and/or dependence is less common among females than among their male counterparts, although under diagnosis may explain at least part of this difference. However, women with schizophrenia may find it more difficult than men to stop using substances, which would explain the greater negative impact of SUD in female patients.

## 4 FIRST-EPISODE PSYCHOSIS

Early phases of psychosis, including at-risk mental states (ARMS) and first episode psychosis (FEP), are considered critical periods for the posterior prognosis of the psychotic disease. Mental health services around the world have developed specific treatment programs that offer integrated interventions. However, the reported gender differences in early phases of psychosis are inconsistent in some cases [56], as we discuss below. Methodological issues, mainly related to sample selection (affective and nonaffective psychosis, age at onset), may help to explain these discrepancies [4].

In general, female patients tend to debut with FEP at older ages than men [2, 4, 5]. In terms of clinical presentation, women tend to present more anxiety and affective symptoms, and parasuicidal behavior; by contrast, male patients tend to present more disorganization and negative symptoms, including social withdrawal, emotional blunting, and amotivation, although the reported findings are not always consistent among studies [9, 57] . With regard to functionality, women tend to have better outcomes in the medium term (*e.g*., three years), but results tend to converge with men after 10 years [32]. Several factors may explain these gender differences, including less comorbid substance use among women and the protective role of certain sex hormones such as estradiol [9].

Substance abuse rates are higher in male patients with FEP [57], with reported prevalence rates of 68-70% for men and 30-48% for women [4, 58, 59]. Compared to women, male FEP patients are more likely to use alcohol, cannabis, and cocaine, and also more likely to use more than one drug [4]. However, these findings must be interpreted cautiously given that SUD may be under diagnosed in female patients [10, 56].

In patients with FEP, SUD comorbidity is associated with poor clinical outcomes, including more severe psychopathology, more positive symptoms and dissociation symptoms, and higher relapse rates [5, 60-63]. Substance use may also be associated with specific distinctive symptoms in FEP, such as depersonalization, derealization and visual or cenestethic hallucinations, as has been described in substance exogenous psychosis [64]. The lower rates of substance use among female patients could be the main reason for better outcomes in women [57] given that studies excluding patients with comorbid SUD have found smaller gender differences [65].

One study found that more than one-third of FEP patients with SUD stopped using the substance in the two years after diagnosis, which was associated with better clinical and functional outcomes [66]. In that study, the lower prevalence of SUD was especially significant for cannabis use, which fell from 42.9% to 27.2% in the first year of follow-up, and to 23.3% in the second year. Unfortunately, the authors did not assess gender differences in the prevalence of SUD, even though such data would be particularly interesting given that women in that study had a lower baseline rate, which means different patterns of use would be expected during follow-up. In addition, some studies have suggested that there may be neurobiological gender differences in the distribution of gray matter in regions thought to be involved in the addiction cycle, and these gender differences could lead to different patterns of cessation, maintenance, and relapse [16]. Ayesa-Arriola and collaborators (2020) found that cannabis use remained more common in males than in females at 10 years of follow-up [32], but gender differences detected in alcohol use at baseline were no longer present. These findings suggest that female FEP patients may experience gender-specific challenges when being treated for SUD, similar to the unique challenges reported in women with SUD [8]. It is important to keep in mind that the aforementioned findings were based only on patients who continued with follow-up (*i.e*., no dropouts) at the early intervention unit, which implies that these patients may have a certain degree of insight that could influence their intention to stop using drugs.

The connection between cannabis use and schizophrenia is supported by strong evidence [67]. In adolescents, cannabis use during neurodevelopment is a risk factor in individuals with a genetic predisposition for schizophrenia [56]. In addition, data show that while any cannabis use increases the risk for psychosis [68], frequent smokers of high-potency cannabis may have a nearly four-fold higher risk of developing psychotic symptoms or a psychotic diagnosis [68, 69]. Di Forti and colleagues (2019) evaluated a cohort of patients with psychotic disorders, most of whom were male [68]. Nearly one-fourth (23%) of the sample refused to participate in the study; interestingly, the refusal rate was significantly higher among women, which could indicate the difficulties that female patients have in discussing their pattern of drug use. Recently, Pence *et al*. (2022) conducted a systematic review to explore gender differences in environmental risk factors and psychosis [70]. Those authors evaluated data from seven studies about the differential gender effect of substance use in psychosis, finding that cannabis could be a stronger risk factor in females since cannabis use is associated with an earlier onset of psychosis [20, 71, 72]. Based on their findings, the authors suggested that the telescoping effect in female patients with addictions could play an important role in this gender difference.

Cannabis use disorder is the most common substance use disorder in FEP [73]. However, cannabis use is less common in female patients with FEP [5, 74-76]. While almost 70% of men with FEP smoke cannabis, the corresponding percentage in women is around 28-36% [4, 58, 59]. Female patients with FEP start smoking cannabis later than men [4, 77, 78]. Interestingly, this difference in age of onset between sexes has not been detected for other drugs, although recent findings suggest that female FEP patients may start smoking cannabis at an earlier age, similar to trends observed for other substances [74]. One study found that even though male patients used more cannabis (both in terms of quantities and frequencies) at baseline and during follow-up, female patients were less successful in reducing their use of cannabis over time [56], perhaps due to more severe withdrawal symptoms [79]. A review of the interaction between gender and cannabis in early phases of psychosis [56] suggested that 17-β-estradiol was a plausible common factor that could explain gender differences in both psychosis and addiction.

Although substance use, especially the use of more than one substance, is associated with earlier age at onset of psychosis [4, 80-85], cannabis use has a particularly strong association with an earlier age of onset of FEP [86-89]. However, it is still not clear whether there are gender differences in this association [4, 72], and some authors have suggested that cannabis use could lower the age of onset of psychosis in females by almost 6 years, but not in male patients [72].

The self-medication hypothesis [90] posits that some individuals use substances to alleviate symptoms, which would explain the use of cannabis in patients with psychosis [91]. The patient’s subjective reason for using cannabis could be an indicator of certain clinical features in that patient. Both psychotic and non-psychotic patients have reported using cannabis for relaxation, sleep, euphoria, or to reduce sadness. By contrast, patients with FEP have reported using cannabis to organize their thoughts and decrease hallucinations [92]. A recent study in patients with FEP found that the most common reason for using cannabis in both males and females was “to relax”, particularly among female patients [74]. While this difference could be attributed to the higher prevalence of affective and anxiety symptoms in female patients with FEP, that study found no association between cannabis use and clinical variables at admission or discharge. Cannabis use was associated with worse functionality in males and better functionality in females. Other studies have found that the clinical presentation of psychosis differs between cannabis and non-cannabis-using male psychotic patients, with more dissociative symptoms [63] and disorganization, and less severe negative symptoms in users, but without differences between female users and non-users [72, 93]. This finding contrasts with the classical perspective, which posits that clinical differences between genders could be due to comorbidities, such as the higher prevalence of SUD among men [9].

In a recent study, Irving *et al.* (2021) sought to determine whether there are gender differences in symptomatology and substance use between FEP patients [94]. That study used natural language processing, an innovative machine learning approach, to analyze a large sample of electronic health record data. On the univariate analysis, several negative and cognitive symptoms (poverty of speech, social withdrawal, poverty of thought) and use of all substances evaluated (amphetamine, cannabis, cocaine and MDMA) were more common in males, while affective symptoms, both manic (elation, pressured speech, mood instability) and depressive symptoms (worthlessness, poor concentration, guilt, poor appetite, low energy, low mood, and tearfulness) were more common in women. When the authors controlled for substance use, negative symptoms remained more prevalent in men while manic and depressive symptoms became even more common in women. Therefore, the higher prevalence of negative symptoms in men is not only due to drug-related amotivational syndrome. By contrast, after controlling for drug use, differences in aggression, agitation, paranoia and grandiosity lost significance, suggesting that differences in these symptoms were mediated by drug use [94].

In short, substance use and SUD are less prevalent in female FEP patients (as occurs in schizophrenia), which could explain the better treatment outcomes in female FEP patients. The onset of FEP in females generally occurs at older ages, potentially due to the protective role of the female sex hormone estradiol [9]; however, this gender-related age gap may be reduced in women who use cannabis. Under the self-medication hypothesis, the different cannabis use patterns observed in women could be attributable to the specific cluster of symptoms (*e.g.,* more anxiety and affective symptoms), which are more common among female FEP patients.

Barajas and collaborators (2015) conducted a comprehensive literature review to evaluate prodromic states of psychosis [95]. As those authors noted, there is no consensus regarding gender differences in at-risk mental states (ARMS), probably due to the limited number of studies in this field. However, that review revealed gender differences in symptomatology, functioning, and social support, indicating that the gender differences in psychosis extend across the different phases of the disease. However, they did not assess gender differences in substance use among ARMS patients. A multicenter, naturalistic prospective study was carried out in Early Detection and Intervention Centers across all of Europe to check for the presence of gender differences in symptoms, drug use, and comorbidities among ARMS patients, who were selected through the Comprehensive Assessment of At-Risk Mental State (CAARMS) criteria [96]. That study found that a higher proportion of male ARMS patients used cannabis and did so more frequently; however, these differences became non-significant after statistical adjustment. In addition, there were no gender differences in the proportion of cannabis-dependent patients, nor were gender differences observed in the use, abuse, or dependence on other drugs such as cocaine, amphetamines, and hallucinogens. Rietschel and collaborators (2017) found that SUD was more common among male ARMS patients, although prevalence rates were not determined for each drug separately [53]. Female ARMS patients showed more comorbidity with affective and anxiety disorders, but less severe negative symptoms and more general psychopathology; nevertheless, all of these differences became non-significant after adjustment for multiple testing [96]. Other studies have found small clinical differences between male and female ARMS patients, with male patients presenting more social withdrawal and concentration problems and females presenting more anxiety and fears [97].

This lack of clear gender differences in the population at-risk for psychosis suggests that differences may be greater among patients with more advanced stages of the illness [9]. Alternatively, the lack of marked differences could be due to small sample sizes, and the fact that male patients are underrepresented in at-risk psychosis samples [98]. Barajas *et al.* (2015) underscored the importance of considering gender differences when assessing risk states for psychosis [95]. For example, positive symptoms have a greater weighting in the Ultra-High Risk (UHR) criteria, which we would expect to facilitate the detection of psychosis in women *versus* men; in addition, women are more likely to seek medical attention.

In short, the available data on gender differences in prodromic states of psychosis are inconsistent, and the potential differential effect of drug use, particularly cannabis, has received scant attention.

## 5 GENDER DIFFERENCES IN SUBSTANCE-INDUCED PSYCHOSIS

Substance-induced psychoses are a heterogeneous group of brief psychotic syndromes triggered by acute intoxication or withdrawal of a psychoactive substance use [99, 100]. Psychotic symptoms can last for several days or weeks after drug use. In some cases, it can be difficult to determine whether psychotic symptoms are substance-induced or indicative of an underlying psychotic disease [1]. In this sense, Martinotti and collaborators (2021) recently discussed some of the controversial criteria of substance-induced psychosis in DSM-5 and CIE-10 and proposed a new categorization called substance-related exogenous psychosis [64]. The authors point out some of the distinctive clinical aspects in substance-induced psychoses: depersonalization, derealization, visual and cenesthetic hallucinations, preserved insight, and, particularly, modification of affection [64, 101]. As we have pointed out, female patients present more affective symptoms in the initial phases of psychosis [9, 94, 96]. Therefore, this symptom should be considered carefully when distinguishing a primary psychosis and a substance-induced disorder in women.

Some patients with substance-induced psychosis will transition to schizophrenia in the following years. However, for some authors distinguishing whether chronic drug use induces a different form of chronic psychotic syndrome or whether it is a schizophrenic disorder is still an unanswered question [64]. According to a recent systematic review and meta-analysis, the proportion of patients with substance-induced psychosis who later transition to schizophrenia is approximately 25% [102]. In that metanalysis, the main predictor of transition was the type of drug. Specifically, cannabis was associated with the highest risk, accounting for 34% of patients. The risk associated with hallucinogens and amphetamines was intermediate while the risk of opioid, alcohol, or sedative-induced psychosis was very low [102]. Other studies have found that half of the patients with cannabis-induced psychosis will develop a schizophrenia-spectrum disorder [103], with higher rates among young males. The cannabis-induced disorder has been identified as a highly unstable diagnosis, whereas a stimulant-induced disorder may have a better prognosis and a lower rate of transition to schizophrenia [104]. A considerable number of novel psychoactive substances have been associated with acute and chronic symptoms of psychosis [105], but we still have too small a case series to estimate transition ratios and differences between genders.

Fiorentini *et al.* found that gender was not a predictor of the likelihood of transition. Nevertheless, those authors also pointed out that most of the studies conducted to date included samples with a higher proportion of males (mean: 61%). A more recent review on this topic did not evaluate gender differences for this type of psychosis [106].

In a Swedish cohort of patients (n = 7606) with a diagnosis of substance-induced psychotic disorder [107], the risk of transition to schizophrenia was only 11%. The associated risks for the substances studied were similar to other studies, with cannabis showing the highest risk. The prediction model developed in that study showed that the main variable associated with a greater risk for transition was male sex, followed by early age at onset, a high familial risk score for non-affective psychosis, and first diagnosis at a specialist or an inpatient care setting.

In recent years, the prevalence of methamphetamine-induced psychosis has increased worldwide, mainly due to the increased use of this drug in many countries. The main risk factors associated with methamphetamine-induced psychosis are as follows: methamphetamine dependence (clinical criteria); history of hospitalization for SUD; duration of use; and, importantly, male sex [108]. Interestingly, drug concentration levels in hair samples have not been clearly associated with the risk for psychosis. Other sociodemographic factors associated with methamphetamine-induced psychosis are being single, being unemployed, having low education, and having childhood adversities [109].

A narrative review of cocaine-induced psychosis found that reported gender differences are inconsistent and often contradictory [110], with some studies showing a higher prevalence among male users [111, 112], and others showing a greater risk for female patients [113].

In short, due to inconsistent data, gender differences in substance-induced psychosis are not clear, but likely to be dependent on the particular drug. In general, males seem to have a higher risk of transitioning to schizophrenia, but other confounding factors should be taken into account.

## CONCLUSIONS AND FUTURE PROSPECTS

In patients with a psychotic spectrum diagnosis, the prevalence of substance use and SUD is lower in women in all phases of the disorder, including the prodromic states of psychosis, first episodes, and during the course of schizophrenia. Although the reasons for this difference are not entirely clear, under diagnosis in female patients could play a role. Importantly, this gender gap appears to be decreasing in recent years.

Women with schizophrenia and comorbid SUD may be more negatively impacted than men by drug abuse, including more difficulties in stopping the use of the substance and greater impairment in functionality. Moreover, the negative factors (such as stigma or self-stigma) that affect women who use drugs are even stronger in patients with psychotic illnesses.

Based on the current available data, it seems clear that treatment programs for psychotic patients should take into account the specificities of female patients with addiction. Future studies are needed to better elucidate the specificities of this group of patients in terms of drug use patterns, treatment response, and prognosis.

## LIST OF ABBREVIATIONS

ARMS: At-risk Mental States
CAARMS: Comprehensive Assessment of At-Risk Mental State
DD: Dual Disorder
DDSI: Dual Diagnosis Screening Interview
FEP: First Episode Psychosis
SUD: Substance Use Disorder
UHR: Ultra-High Risk
